# An Eigenspace approach for detecting multiple space-time disease clusters: Application to measles hotspots detection in Khyber-Pakhtunkhwa, Pakistan

**DOI:** 10.1371/journal.pone.0199176

**Published:** 2018-06-19

**Authors:** Sami Ullah, Hanita Daud, Sarat C. Dass, Hadi Fanaee-T, Alamgir Khalil

**Affiliations:** 1 Department of Fundamental & Applied Sciences, Universiti Teknologi PETRONAS, Bandar Seri Iskandar, Tronoh Perak, Malaysia; 2 Department of Biostatistics, University of Oslo, Oslo, Norway; 3 Department of Statistics, University of Peshawar, Peshawar, Pakistan; Johns Hopkins Bloomberg School of Public Health, UNITED STATES

## Abstract

Identifying the abnormally high-risk regions in a spatiotemporal space that contains an unexpected disease count is helpful to conduct surveillance and implement control strategies. The EigenSpot algorithm has been recently proposed for detecting space-time disease clusters of arbitrary shapes with no restriction on the distribution and quality of the data, and has shown some promising advantages over the state-of-the-art methods. However, the main problem with the EigenSpot method is that it cannot be adapted to detect more than one spatiotemporal hotspot. This is an important limitation, since, in reality, we may have multiple hotspots, sometimes at the same level of importance. We propose an extension of the EigenSpot algorithm, called Multi-EigenSpot that is able to handle multiple hotspots by iteratively removing previously detected hotspots and re-running the algorithm until no more hotspots are found. In addition, a visualization tool (heatmap) has been linked to the proposed algorithm to visualize multiple clusters with different colors. We evaluated the proposed method using the monthly data on measles cases in Khyber-Pakhtunkhwa, Pakistan (Jan 2016- Dec 2016), and the efficiency was compared with the state-of-the-art methods: EigenSpot and Space-time scan statistic (SaTScan). The results showed the effectiveness of the proposed method for detecting multiple clusters in a spatiotemporal space.

## Introduction

Detection of spatiotemporal disease clusters plays a fundamental role in epidemiology and public health. Health organizations collect data on the reported disease cases and the population at risk for different sub-regions over a range of time-points (years or months) to conduct surveillance. Using this spatiotemporal data, detecting the abnormally high-risk regions in the spatiotemporal space assists the health officials to identify their target of interest for possible interventions. In this situation, a disease cluster is defined to be a sub-region or a group of sub-regions in a spatiotemporal space where the observed case counts are unusually higher than what is to be expected if no cluster exists [1,2]. The detection of such space-time clusters guides the epidemiologic research to find the environmental factors that possibly affect the spread of disease in an area.

A number of statistical methods have been developed for detecting regularly shaped (circular, square or rectangular) space-time clusters [3–8]. In certain situations, where the disease cases tend to bunch up in the irregularly shaped areas due to some irregular features in the terrain, these methods are not practically feasible to detect clusters with real shapes. This study has mainly focused on detecting space-time clusters with arbitrary shapes and sizes. A few algorithms have been developed to detect space-time clusters with irregular shapes. A flexible space-time scan statistic [9], a grid-based method [10,11], and a space-time permutation scan statistic [12] were proposed to detect space-time clusters with irregular shapes. All of the algorithms for disease cluster detection discussed above, are associated with the strong parametric model assumptions (e.g. Poisson or Gaussian counts) [13], which limit their applicability for some nontraditional data sources where these assumptions are not met. Addressing this problem, an Eigenspace-based algorithm called EigenSpot was recently proposed [14] to detect space-time clusters with no restriction on the distribution and quality of the data or the shape of the cluster. However, this method can detect single hotspot only and is unable to detect multiple clusters. In the disease surveillance when one cluster is detected, it is of interest to know if there are additional clusters of high-risk regions present in the spatiotemporal space.

This research has aimed to propose an extension of the EigenSpot algorithm called the Multi-EigenSpot to allow for detecting multiple clusters in a spatiotemporal space. Our proposed algorithm uses the spatiotemporal matrix of expected disease cases as the baseline information instead of the population matrix in the EigenSpot. Using the expected case matrix as the baseline information, we can replace the observed cases by the respective expected cases for the previously detected regions in the spatiotemporal space and re-run the algorithm to detect additional clusters, if exist.

For visualizing the clusters, the most widely used visualization tool called heatmap was linked to the proposed method through an additional matrix of Relative Risk (RR). Heatmap is a graphical representation of a data matrix, which uses colors to communicate relationships between data values that would be much harder to understand if presented numerically in a spreadsheet. It has important applications in the spatiotemporal data analysis. For example, in [15], the heatmap was used for spatiotemporal pattern recognition in the average daily temperature data, and in [16], for visualization of the space-time clusters in the malaria case data. Heatmap visualization of the high-risk clusters can be helpful to conduct the real-time and online surveillance.

The Eigenspace-based methods are inherently different from the scan statistic-based methods and, subsequently, have different applications. The Eigenspace-based algorithms identify space-time disease hot spots by tracking the changes in the space-time occurrence structure instead of an exhaustive search over the space like the scan statistic-based methods. Scan statistics-based methods are more useful for sensitive applications when the assumptions about the distribution of the data and nature of the cluster are satisfied. However, for some nontraditional data sources, where these assuumptions are not met, the Eigenspace-based method is an ideal solution for detecting the potential clusters in a spatiotemporal space with no restriction on the distribution and quality of the data, or shape of the cluster. The Eigenspace-based methods detect the clusters of homogeneous regions in terms of a disease occurrence structure and do not restrict the regions in a cluster to be the spatial neighbors. Since our proposed algorithm is based on the EigenSpot method, before presenting the proposed algorithm, a brief review of the EigenSpot method is given in the following section.

### EigenSpot algorithm

The inputs of the EigenSpot algorithm are two spatiotemporal *m* × *n* matrices, *C*, the reported disease cases and, *B*, the baseline information (population at risk) where *m* represents the number of regions and *n* represents the number of temporal instants. Each cell in each matrix represents a count corresponding to a specific region and time. Given these matrices, the EigenSpot algorithm aims to identify a subgroup of regions in the spatiotemporal space where the reported cases are unexpected with respect to the baseline information. Each matrix is decomposed using a one-rank SVD to obtain the principal left and principal right singular vectors. The elements of the principal left singular vectors are associated to the spatial dimension and the principal right-singular vectors to the temporal dimension. In the next step, the distances between the corresponding elements of the pair singular vectors are calculated. If the spatial singular vector for the population matrix is represented with (*sb*_1_, *sb*_2_, …, *sb*_*n*_) and for the case matrix with (*sc*_1_, *sc*_2_, …, *sc*_*n*_), then the subtract vector is calculated as:

$$ds\;=\;[{ds}_{1}\;=\;{sc}_{1}-{sb}_{1}\;{ds}_{2}\;=\;{sc}_{2}-{sb}_{2}\ldots \;{ds}_{n}\;=\;{sc}_{n}-{sb}_{n}]$$

Similarly, for the temporal dimension, the subtract vector is given by:

$$dt\;=\;[{dt}_{1}\;=\;{tc}_{1}-{tb}_{1}\;{dt}_{2}\;=\;{tc}_{2}-{tb}_{2}\ldots \;{dt}_{m}\;=\;{tc}_{m}-{tb}_{m}]$$

A z-score control chart is applied to vectors *ds* and *dt* with a significance level *α*, to identify the out of control spatial and temporal components. The locations of hotspot regions in the spatiotemporal space are approximated by the joint combination of the out of control spatial and temporal components.

## Methods

For our proposed algorithm, we consider the situation in which the data on the observed disease cases and the population at risk are aggregated for different sub-regions over a range of discrete time-points (month, or year). In our proposed algorithm, the data on the disease counts and the population at risk are arranged in the form of identical *m* × *n* spatiotemporal matrices, *C* and *P*, respectively, where *m* denotes the number of components in the spatial dimension (sub-regions) and *n* the number of components in the temporal dimension (time-points). Given the spatiotemporal matrices, *C*, (observed cases) and, *P*, (population at risk), two spatiotemporal matrices, *E* (expected cases) and, *R* (relative risks) are calculated. For the expected disease cases, if no cluster exists in the spatiotemporal space, we use the formula proposed in [17], which assumes the reported cases to be distributed over the spatiotemporal space proportional to the respective population count. The risk measure, RR, is the most widely used measure of disease incidence, which is calculated as the proportion of the observed cases to the expected cases [18]. The Singular Value Decomposition (SVD) is applied on each matrix, *C* and *E*, and the left and right singular vectors are calculated. The singular value decomposition of a spatiotemporal *m* × *n* matrix, *M* is of the form *M* = *UDV*^*t*^, where the columns of *U* are the left singular vectors corresponding to the spatial dimension, and the columns of *V* are the right singular vectors corresponding to the temporal dimension. *D* is a diagonal matrix whose diagonal entries are the Eigenvalues of matrix, *M*. For the purpose of comparison, only the singular vectors corresponding to the largest Eigenvalue were considered because these principal vectors explain or extract the largest part of the inertia of the data table [19]. If we assume *C* and *E* are identical, then their principal left and right singular vectors are identical as well, i.e. the corresponding elements in the pair singular vectors stay in a zero distance. If some change occurs in *C*, this change can be detected from the changes in the principal singular vector’S elements. In such cases, some distances between the corresponding elements of the pair singular vectors become abnormal for the components corresponding to the affected areas in both the spatial and temporal dimensions. Our approach uses the z-control chart for monitoring the distances between the corresponding elements of the pair singular vectors. The corresponding elements of the pair left singular vectors showing abnormal difference are associated to the spatial components of a cluster, and of the pair right singular vectors to the temporal components. If the abnormal components are found in both dimensions, matrix *C* is upgraded by replacing the elements (observed cases) corresponding to the out of control spatial and temporal components by the respective expected cases. In addition, matrix, *R* is upgraded by replacing the elements corresponding to the out of control components by their average value to further visualize these elements on the heatmap with the same color as a hotspot. The process is repeated with the upgraded matrix *C* and the original matrix of the expected cases, *E*. The matrices, *C* and *R*, are upgraded iteratively until no out of control component is found in either spatial or temporal dimension. Since the upgraded elements in matrix *R* are used to approximate the hotspots, the elements in the upgraded matrix *R*, other than the average values, are replaced by 1 to differentiate the upgraded elements from the other non-upgraded ones. The resulting matrix *R* is then visualized on the heatmap showing the different average relative-risks with different colors. In case no space-time cluster exists, the resulting heatmap will have all elements equal to 1 showing a dark-blue color only. Different colors on the heatmap other than dark-blue approximate different space-time clusters. The sub-regions in a cluster are homogeneous with respect to the space-time occurrence structure and represented by a same color on the heatmap.

**Fig 1:**
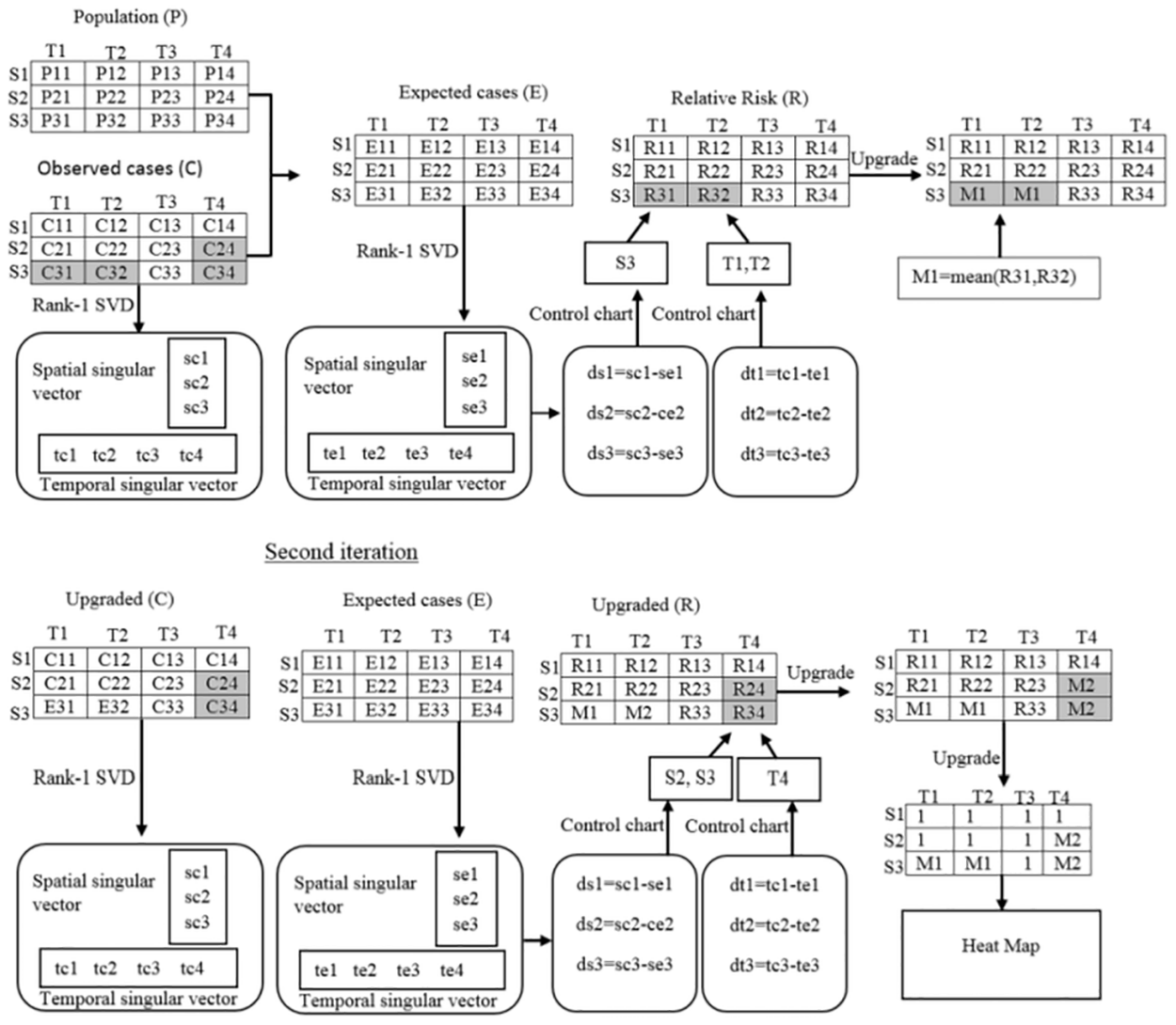
Example demonstrating the proposed algorithm.

Fig 1 shows an illustrative example of how our proposed algorithm can detect multiple hotspots in a spatiotemporal space. Given the 3×4 spatiotemporal matrices of the observed cases, *C*, and the population, *P*, such that *C*_*ij*_ and *P*_*ij*_ represents the observed cases and population, respectively, for the *i*^*th*^ region (*S*_*i*_) over the *j*^*th*^ time-point (*T*_*j*_). The two shaded areas in the case matrix in Fig 1 are the two different clusters of interest to be detected by the proposed method. The conjunction of the third row with the first-second columns represents the first likely cluster and the conjunction of the second-third rows with the forth column represents the second (additional) cluster. Since there are two clusters existing in the data, the proposed algorithm needs to be iterated two times. The first iteration will result in two similar values equal to *M1* in the upgraded matrix *R*, approximating the first likely cluster. The first likely cluster is removed by replacing the observed cases by the corresponding expected cases and the process is iterated to search for an additional cluster. The second iteration will results in two other similar values equal to *M2* in matrix *R*, approximating the additional cluster. The elements in the last upgraded matrix *R*, other than *M1* and *M2*, are replaced by 1 and visualized on the heatmap. The heatmap visualizes these two clusters with different colors.

### Multi-EigenSpot algorithm

In this section, our proposed algorithm is presented in more detail. This algorithm requires three types of tools: 1) SVD for finding the singular vectors of a non-square matrix, 2) a statistical process control tool for monitoring distances between the corresponding elements of the pair singular vectors and 3) a visualization tool (heatmap) for visualizing the detected clusters. The detailed stepwise process and how these techniques are deployed in the algorithm is given below.

**Step-1**: Calculate the spatiotemporal matrices of the expected disease cases and relative risks.
  Given the spatiotemporal data matrices, *C* (observed disease cases) and, *P* (population), the expected number of disease cases for the *i*^*th*^ region over the *j*^*th*^ time-point, *E*_*ij*_ is calculated as:
  $$E_{ij}\;=\;\frac{{C.}_{j}}{{P.}_{j}}\times \;P_{ij}$$ (1)
  $$E\;=\;\left[\begin{array}{ccc} E11 & \ldots & E1n\\ \vdots & \ddots & \vdots \\ Em1 & \ldots & Emn \end{array}\right]$$
  where *C*._*j*_ is the *j*^*th*^ column-total of matrix, *C* representing the total observed cases of a particular disease in the whole study area over the *j*^*th*^ time-point; *P*._*j*_ is the *j*^*th*^ column-total of matrix, *P* representing the total population of the whole study area over the *j*^*th*^ time-point; *P*_*ij*_ is the population of an *i*^*th*^ sub-region over the *j*^*th*^ time-point.
  The RR for the *i*^*th*^ sub-region over the *j*^*th*^ time-point is calculated as:
  $$R_{ij}\;=\;\frac{C_{ij}}{E_{ij}}$$ (2)
  $$R\;=\;\left[\begin{array}{ccc} R11 & \ldots & R1n\\ \vdots & \ddots & \vdots \\ Rm1 & \ldots & Rmn \end{array}\right]$$
  Calculating matrix *R*, our goal is to visualize the clusters on the heatmap.
**Step-2**: SVD of matrices, *C* and *E*.
  The one-rank SVD is used to obtain the principal left and right singular vectors for each matrix, *C* and *E*. Our approach requires only the principal singular vector corresponding to the highest Eigenvalue. Because the first principal singular vector accounts for the majority of variance in the data [19]. For matrix, *C*, let’s denote the principal left singular vector with *SC* = (*sc*_1_, *sc*_2_, …, *sc*_*m*_) and the principal right singular vector with *TC* = (*tc*_1_, *tc*_2_, …, *tc*_*n*_). Similarly, for matrix, *E*, the principal left singular vector is denoted by *SE* = (*se*_1_, *se*_2_, …, *se*_*m*_) and the principal right singular vector by *TE* = (*te*_1_, *te*_2_, …, *te*_*n*_)
  The elements in the principal left singular vectors are associated to the components in the spatial dimension, and in the principal right singular vectors to the components in the temporal dimension.
**Step-3**: Calculate the subtract vectors.
  Calculate the subtract vector of the pair left singular vectors as:*DS* = *SC* − *SE*, and that of the pair right singular vectors as: *DT* = *TC* − *TE*.
**Step-4**: Identify abnormally higher distances in the corresponding elements of the pair singular vectors.
  Calculate the standardized vectors of z-scores from the subtract vectors *DS* and *DT* and apply the z-score control chart on both vectors with the level of significance *α*. Those elements of the subtract vectors *DS* and *DT* that obtain a left-tailed p-value less than *α* are considered out of control. The out of control elements in *DS* and *DT* are associated to the abnormal components in the spatial and temporal dimensions, respectively [14].
**Step-5**: Upgrade matrices, *C* and *R*.
  If the abnormal components are found in both vectors *DS* and *DT*, upgrade matrix, *C* by replacing the elements corresponding to the abnormal spatial and temporal components, by the respective expected cases. Similarly, matrix R is upgraded by replacing the elements corresponding to the abnormal spatial and temporal components by their average value.
**Step-6**: Find additional abnormal components in the spatial and temporal dimension.
  Repeat Steps (2–5) until no abnormal component is found in each dimension.
**Step-7**: The elements in the last upgraded matrix *R*, corresponding to the components (spatial/temporal) which were not found to be abnormal, are replaced by 1.
**Step-8:** Visualize the resulting matrix *R* on the heat map on which the average RR-values are represented by different colors.

Hotspots: The colored regions on the heatmap corresponding to different average RR-values (greater than 1) show multiple space-time hotspots. If no cluster exists, then all the data values on the heatmap will be equal to 1, showing a dark-blue color only.

**Fig 2:**
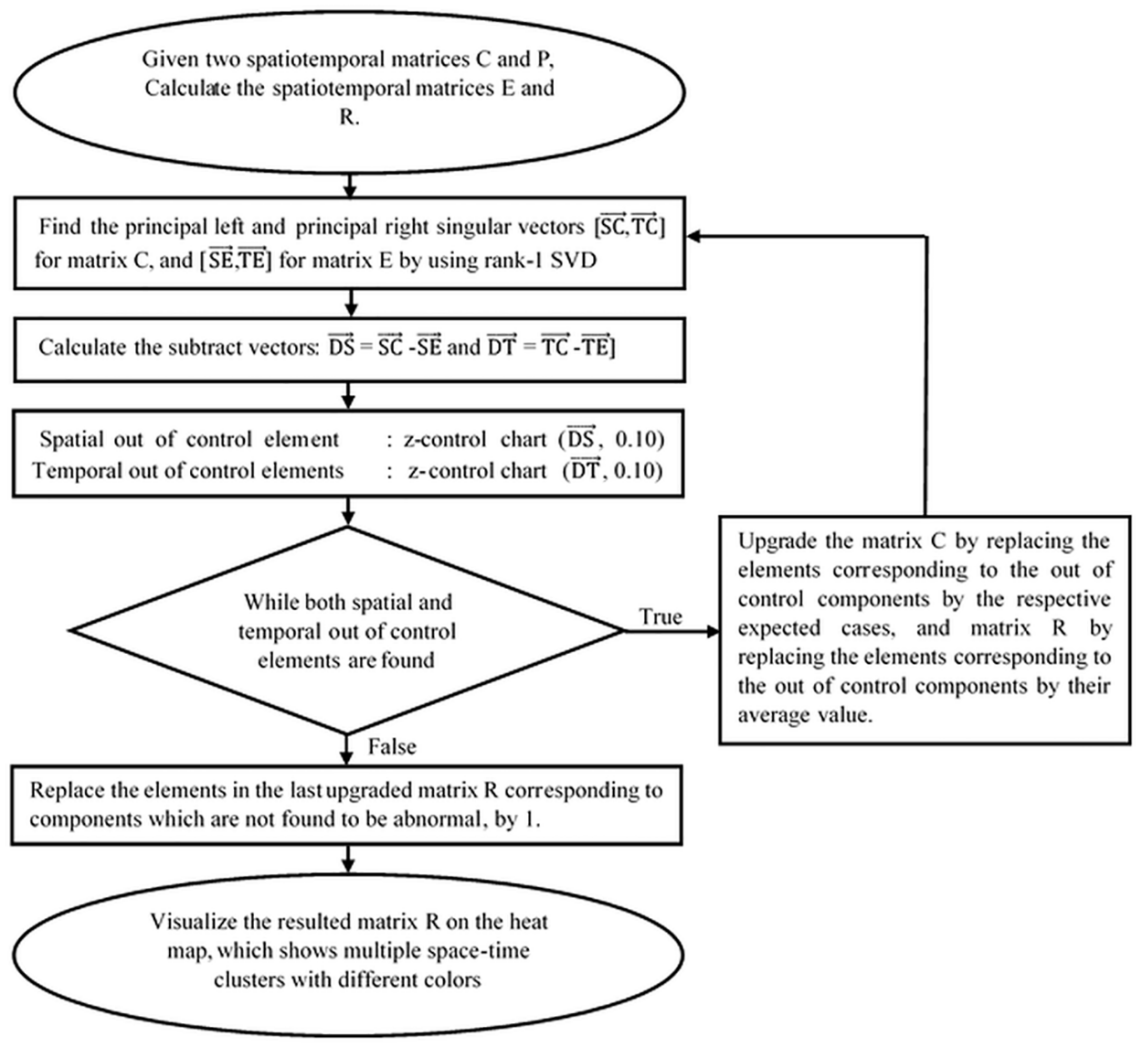
Methodology flowchart.

The proposed method is explained in the flowchart given in Fig 2. The software solution in MATLAB is given in the S1 File.

## Results and discussion

### Measles case study in Khyber-Pakhtunkhwa, Pakistan

Measles is a communicable viral disease characterized by a cough, fever and maculopapular rashes. The World Health Organization’s (WHO) measles data showed 67,524 confirmed measles cases all over the world in the year 2015 and 16,846 till 13th June 2016 [20]. In Pakistan measles is a leading cause of death among young children due to the low vaccination coverage [56%-88%], which varies over different districts nationwide [21,22]. A case-based surveillance system is functional in the targeted areas of the country [23], but an improved surveillance system is still needed to devise the measles control strategies. Detecting space-time measles clusters assists identifying the regions of deprivation for the timely interventions of Public Health organizations.

**Fig 3:**
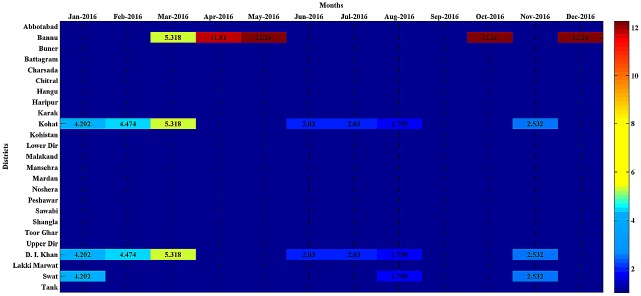
Heatmap.

**Fig 4:**
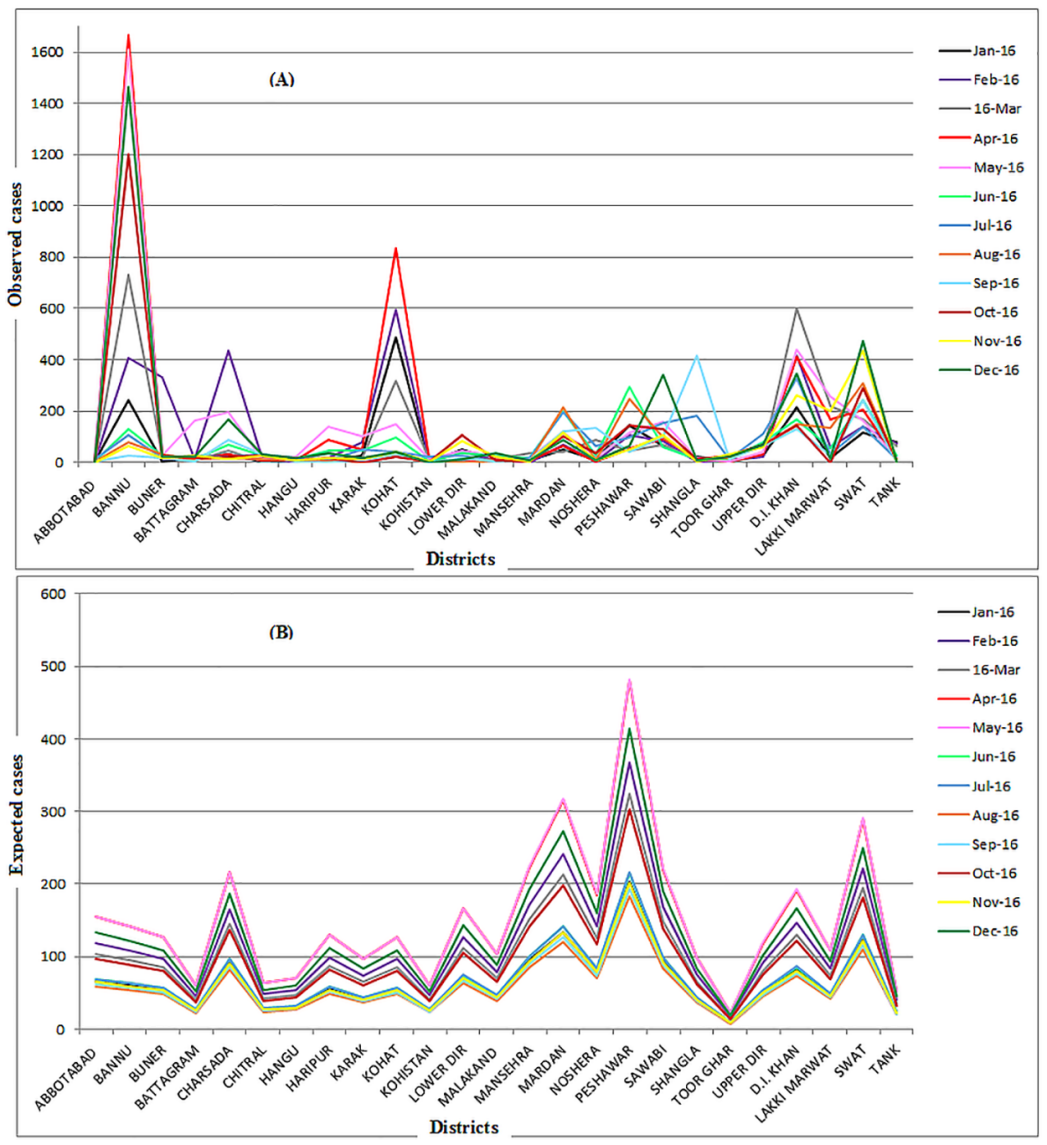
(A) The observed measles cases, (B) The expected measles cases.

The total area of Pakistan is divided into five provinces (Sindh, Balochistan, Punjab, Khyber-Pakhtunkhwa, and Gilgit-Baltistan). The proposed method was applied to detect the high-risk spatiotemporal clusters of measles cases in the Khyber-Pakhtunkhwa province during (Jan 2016-Dec 2016). Khyber-Pakhtunkhwa, the northwestern province of Pakistan, shares a very long border with the neighboring country of Afghanistan. The total area of this province is 74,521 km^2^ with a population of 30.52 million according to the census-2017. The total land area of the province is divided into twenty-five districts. The monthly data on suspected measles cases for each of the twenty-five districts over the months Jan 2016-Dec 2016 were collected from the website of the District Health Information System (DHIS), Khyber-Pakhtunkhwa [24]. The details to access the data were publically available in the quarterly report-2016 [25]. All hospitals in a district report the registered measles cases to the respective district office of DHIS on a monthly basis, and these offices further report the monthly data to the provincial DHIS office. The district wise population data of Khyber-Pakhtunkhwa for the year 2016 is available publically in [26]. The Population at risk was assumed to be constant during Jan 2016 to Dec 2016. The collected data on suspected measles cases and the population at risk are available in the S2 File. Based on these spatiotemporal data sets, the proposed algorithm with *α* = 0.10 generated a heatmap showing four space-time clusters with different colors (Fig 3). The threshold alpha was set to 0.10 because in such frequent diseases, where most of the regions contain the observed cases higher than the expected cases, setting alpha to 0.05 or 0.01 may miss many high risk regions or may detect no hotspot. The observed measles cases and expected measles cases for each of the twenty-five districts over each month (Jan 2016-Dec 2016) were displayed on the graph in Fig 4A and 4B, respectively, to validate the results.

The resulting heat map in Fig 3 showed that the first likely measles cluster occurred in the district of Bannu for the months (May, Oct, Dec) with an average RR = 12.26, represented with a dark-red color, while the second likely cluster occurred in the same district for the month of Apr-2016 with an average RR = 11.81, represented with a light-red color. The three districts (Bannu, Kohat and D. I. Khan) appeared as the third likely cluster for the month of Mar-2016 with the average RR = 5.318, denoted by a yellow color. The forth likely cluster occurred in two districts (Kohat and D.I. Khan) for the month of Feb-2016 with an average RR = 4.474, denoted by a light cyan color on the heatmap. The fifth likely cluster occurred in the districts of Kohat, D. I. Khan and Swat for the month of Jan-2016 with an average RR = 4.202 represented with a dark-cyan color. These three districts appeared as a sixth likely cluster for the month of Nov-2016 with an average RR = 2.532. The seventh likely cluster occurred in the districts of Kohat and D. I. Khan for the months (June, July) with an average RR = 2.03. The three districts (Kohat, D. I. Khan and swat) appeared as an eighth possible cluster for the month of Aug-2016 with an average RR = 1.758. The data values equal to 1 on the heatmap, represented by a dark blue color, showing the regions where the burden of measles cases was not found to be abnormal. The sub-regions belonging to one cluster are homogeneous in space-time occurrence structure. The heatmap showed that the measles had highly affected the four districts (Bannu, Kohat, D. I. Khan and swat) during the year, 2016. These districts appeared as the likely clusters periodically at various months suggesting them to be the alarming measles hotspots. It is evident from Fig 4 that all the hotspots regions in the spatiotemporal space exhibited a higher concentration of the observed measles cases than the expected cases and, hence, validated the results of the proposed method.

**Fig 5:**
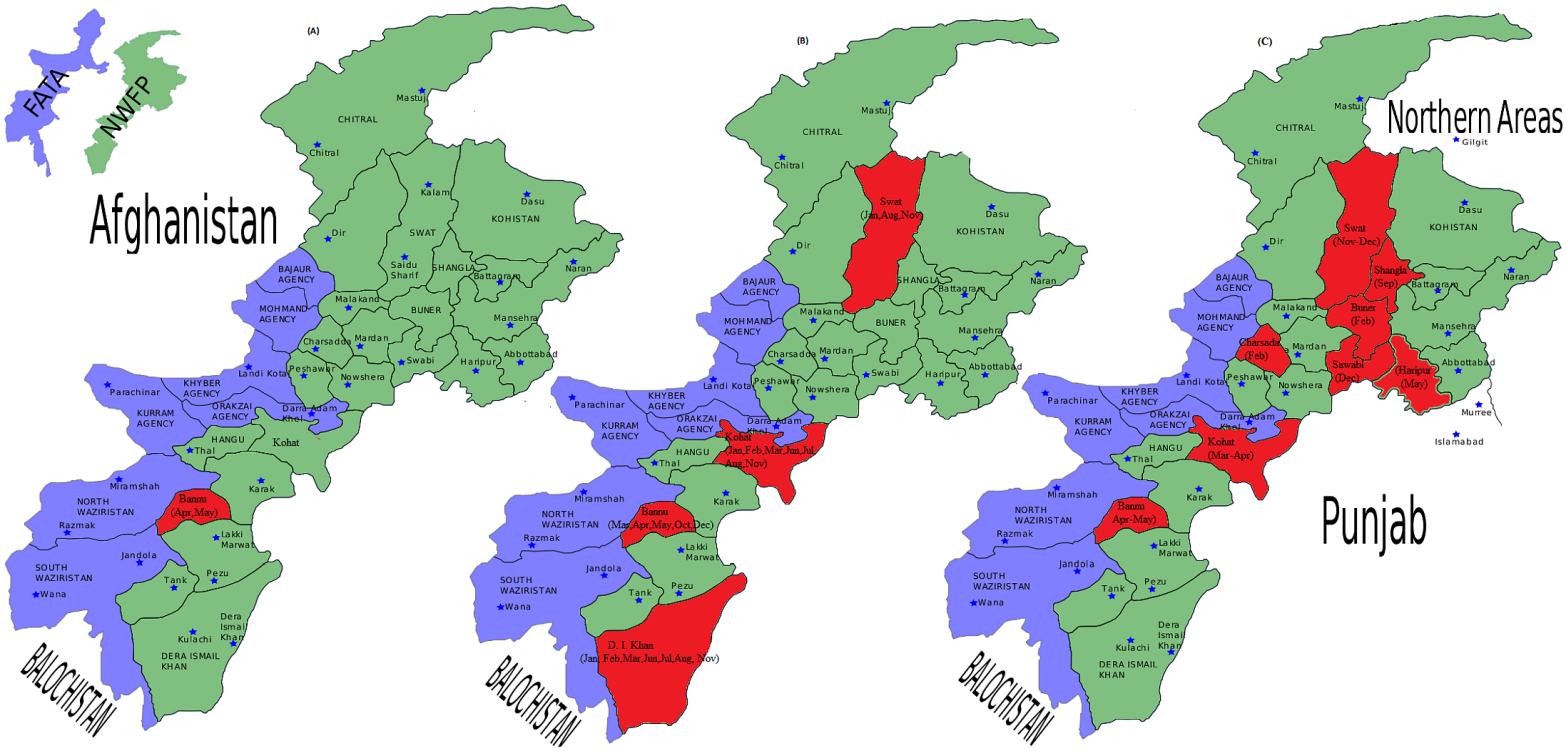
The locations of the clusters detected by (A) EigenSpot, (B) Multi-EigenSpot and (C) SaTScan, respectively.

Most of the hotspots districts detected by the proposed method are the spatial neighbors of the Federally Administered Tribble Areas (FATA) (Fig 5B) and hosting a large number of Internally Displaced People (IDP) from the neighboring FATA regions due to the military operation against terrorism since 2014. The vaccination rate in the IDP camps in the district of Bannu was found to be very low [27], which may have caused the measles outbreaks in the hosting districts because the measles outbreak in the Sindh province during the year 2012 was also attributed to poor vaccination coverage [21]. The heavy influx and continued presence of IDP in these districts may also put a measles case burden in these districts.

### Performance evaluation

The efficiency of the proposed algorithm was compared with the EigenSpot [14], and space-time scan statistic [4] on the measles clusters detection in Khyber-Pakhtunkhwa, Pakistan during the year 2016. These three methods were implemented on the real-world data (S2 File). For the purpose of comparison, the geographical locations of the clusters detected by EigenSpot, Multi-EigenSpot, and SaTScan were shown with a red color in Fig 5A, 5B and 5C, respectively. EigenSpot and Multi-EigenSpot were implemented in MATLAB (R2014a) with a significance level of *α* = 0.10. The space-time scan statistic was implemented with the Poisson model for retrospective analysis in SaTScan [28]. The maximum spatial cluster size was set to 20% of the population at risk, and the maximum temporal cluster size to 20% of the study period, and the high rate clusters were restricted to have a relative risk greater than 1. The SaTScan provided a result-file which showed the names of the counties in each cluster along with the time period for which the cluster persisted. The outputs of each method were summarized in Table 1.

**Table 1:** Performance comparison of EigenSpot, Multi-EigenSpot, and SaTScan.

Method	The detected clusters
EigenSpot	01 (Bannu, Apr, May)
Multi-EigenSpot	08 (Bannu, May, Oct, Dec), (Bannu-Apr), (Bannu, Kohat, D.I,Khan, March), (Kohat, D.I.khan, Feb), (Kohat, D.I.Khan, Swat, Jan), (Kohat, D.I.Khan, Swat, Nov), (Kohat, D.I.Khan, Jun,Jul), (Kohat, D.I.Khan, Swat, Aug).
Space-time Scan statistic	08 (Bannu, Apr-May), (Kohat, Mar-Apr), (Shangla, Sep), (Swat, Nov-Dec), (Buner, Feb), (Charsada, Feb), (Sawabi, Dec), (Haripur, May).

Table 1 showed that EigenSpot detected one likely cluster while our proposed Multi-EigenSpot detected eight likely clusters. The space-time scan statistic detected eight likely clusters including more districts than our proposed approach. SaTScan detected the district of Bannu as a most likely cluster for the months (Apr-May). The proposed method detected the same district (Bannu) in multiple clusters for the months (Mar, Apr, May, Oct and Dec) covering the time-interval of the SaTScan with the additional months (Mar, Oct and Dec). Similarly, the (Kohat, Mar-Apr) was detected as a secondary cluster by the SaTScan while the proposed method detected Kohat in multiple clusters for the months (Jan, Feb, Mar, Jun, Jul and Aug). Moreover, SaTScan detected the district of swat for two months (Nov-Dec) while the proposed approach detected this district in multiple clusters for the months (Jan, Nov and Aug) covering the one month (Nov) of the SaTScan’s output. It is obvious from the results that the proposed approach has approximated the important parts of the SaTScan’s outputs (Fig 5), showing its effectiveness for approximating the significant portion of the ground truth. It is important to note that the direct comparison of SaTScan with Multi-EigenSpot is not an “apple to apple” comparison. Because each of them has its own special characteristics as well as applications. SaTScan is considered as a golden standard method. But it is associated with the strong parametric model assumptions (e.g. Poisson or Gaussian counts) which limit its applicability for some nontraditional data sources where these assumptions are not valid. For example, data from emerging information technology (e.g., internet search engines, social media, smart wearables and smart environmental devices), remote sensing technology (e.g. satellite imaging), over the counter drugs sales, and school health surveys are nontraditional data sources for public health surveillance [29], which may not always follow the assumptions regarding the parametric model and quality of data. In such scenarios, Multi-EigenSpot serves as a nonparametric solution for hotspots detection in the spatiotemporal space. The results showed that the proposed approach has detected some districts repeatedly in various clusters. This is an important advantage of the proposed approach, because identifying such a periodic homogenity in space-time occurrences is of high significance for the epidemiologist to find the possible factors affecting the disease spread in an area. In addition, Multi-EigenSpot is a shape-free approach and does not search for specific shape clusters. Certain diseases are linked to the rivers, forests or roadways which affect the regions along the river, forest or roadways making the extremly irregularrly shaped clusters. In such applications, the proposed approach could be a useful solution for clusters detection regardless of their shapes.

## Conclusion

In this study, we extended the existing EigenSpot algorithm to allow for detecting multiple disease clusters in a spatiotemporal space. The spatiotemporal analysis of the real world data on measles cases proved that the proposed method has addressed the two main limitations (multiple cluster detection and visualization) of the existing EigenSpot algorithm. It is obvious from the results that the proposed approach can approximate the significant portion of the SaTScan’s outputs, showing it to be a useful nonparametric solution when the assumptions of SaTScan regarding the data distribution are not met. In most of the situations, when the data follow the parametric model assumptions, SaTScan is a more efficient solution to the cluster detection problem.

In certain situations, especially in rare diseases where most of the cells in the case matrix are zero, if a space-time region having zero cases is wrongly detected, then it cannot be removed in the following iteration. This is because, replacing the zero observed cases with non-zero expected cases makes it an abnormal region and it can possibly be redetected iteratively and, hence, influences the efficiency of the proposed method. Due to this limitation, the proposed algorithm is recommended for frequent disease cases; and for rare diseases, its results may not always be valid. Future work is required to adjust this algorithm for rare diseases and improve its efficiency for different situations by using a full-rank SVD and different control charts in the algorithm. Moreover, this algorithm is based on the disease cases aggregated for the sub-regions over a discrete time-point, which may miss some clusters that overlap either in space or in time. For example, if an outbreak occurs on the border of a sub-region it may affect a part of the neighboring region, or if a cluster occurs in January and exists until the mid of February.

## Supporting information

S1 File The software solution for the proposed algorithm in MATLAB. (PDF)

S2 File The data on suspected measles cases and the population at risk. (PDF)
