# Supplementary material for: An Eigenspace approach for detecting multiple space-time disease clusters: Application to measles hotspots detection in Khyber-Pakhtunkhwa, Pakistan
Source: PLoS One. 2018 Jun 19;13(6):e0199176. doi: 10.1371/journal.pone.0199176 (PMC6007829; doi:10.1371/journal.pone.0199176)
Supplement: S1 File — (PDF) [file pone.0199176.s001.pdf]

Algorithm: Multi-EigenSpot (Software solution in MATLAB)

//m: number of items in the spatial dimension

//n: number of items in the temporal dimension

//C: an m×n matrix of observed cases

//P: an m×n matrix of the population at risk

//E: an m×n matrix of expected cases

//R: an m×n matrix of relative risk

// α: a threshold value for z-control chart

Input: C, P, α

Output: Heatmap showing multiple clusters

```
1.  sum1 = sum(C); //Calculating matrices E and R

    sum2 = sum(P);

    for  i =1 : m
      for  j = 1:n
        E(i, j) = (sum1(:, j)/sum2(:, j))*P(i, j);

        R(i, j) = C(i, j)/E(i, j);

     end

  end

2.   [U1, S1, V1] =  svd (C)    //  Singular vectors
     [U2, S2, V2] =  svd (E)

3.  DS = U2(:,1)-U1(:,1);       //subtract vectors
    DT = V2(:,1) - V1(:,1);

4.  zs = zscore(DS);                // control chart
    zt = zscore(DT);
    ps = normcdf(zs,'upper');   // p-value
    pt = normcdf(zt,'upper');
```

5. [A1, c] = averagevalue (R, ps, pt)   // average value of R elements corresponding to the //out of control components

```
A = [];
```

6.

```
Kk = 0;
w = m×n;
While c>0 && kk<w
 A = [A A1];
[O, R] = update (C, E, A1, R, ps, pt) // Upgrading C and R
 [U1, S1, V1] =  svd (C)    //  Singular vectors
 [U2, S2, V2] =  svd (E)
 DS = U2(:,1)-U1(:,1);       //subtract vectors
 DT = V2(:,1) - V1(:,1);
 zs = zscore(DS);                // control chart
 zt = zscore(DT);
 ps = normcdf(zs,'upper');   // p-value
 pt = normcdf(zt,'upper')
 kk=kk+1;
 [A1, c] = averagevalue (R, ps,pt)
End
```

7

```
for  i =1 : m              // Replacement of  upgraded R elements other than average values
   for  j = 1:n              // by 1.
      if  (R (i, j)  ≠ A(1, :)
           R (i, j) = 1;
      else
           R (i, j) = R (i, j);
     end
  end
end
```

```
heatmaptext (R)          // Visualization on the heatmap
```

The functions averagevalue() , update() and heatmaptext() used in the algorithm are given below:

```
function [M, c] = averagevalue(R,ps,pt)      //average value of R elements corresponding to out
                                              //of control components
M=0;
c=0;
k=0;
for i=1:m
  for j=1:n
      if  ps < α && pt < α
          t=R (i, j);
          k=k+1
      else
          c=c+1;
         end
  end
end
if   c == m×n
     c = 0;
else
c=1;
     M=mean (t);
end
end

%%%%%%%%%%%%%%%%%%%%%%%%%%%%%%%%%%%%%%%%%%%%%%%%%
function [d, q]=Update(C, E, A ,R, ps, pt)     // Upgrading C and R
k=1;
for i =1:m
      for j = 1:n
          if  ps < α && pt < α
             d(i, j) = E(i, j);
             q(i, j) = A;
             k = k+1;
          else
           d(i, j) = C(i, j);
```

```
            q(i, j) = R(i, j);
        end
    end
end
```

%%%

The source code file for heatmaptext () is available at:
//https://www.mathworks.com/matlabcentral/fileexchange/15877-heat-maps-with-text
