# Supplementary material for: An Eigenspace approach for detecting multiple space-time disease clusters: Application to measles hotspots detection in Khyber-Pakhtunkhwa, Pakistan
Source: PLoS One. 2018 Jun 19;13(6):e0199176. doi: 10.1371/journal.pone.0199176 (PMC6007829; doi:10.1371/journal.pone.0199176)
Supplement: S2 File — (PDF) [file pone.0199176.s002.pdf]

			Observed Measles cases											
S.No.	Districts	Population at risk	Jan-16	Feb-16	Mar-16	Apr-16	May-16	Jun-16	Jul-16	Aug-16	Sep-16	Oct-16	Nov-16	Dec-16
1	ABBOTABAD	1215000	2	4	4	2	4	5	13	5	4	0	0	0
2	BANNU	1110000	241	406	731	1668	1608	131	108	79	24	1200	65	1464
3	BUNER	994000	3	331	24	23	24	25	18	32	17	26	19	20
4	BUTTAGRAM	475000	17	14	7	14	161	14	26	13	5	17	27	23
5	CHARSADA	1696000	33	433	45	31	195	69	22	18	87	20	13	168
6	CHITRAL	497000	4	18	1	8	12	28	30	12	33	26	24	31
7	HANGU	556000	0	0	6	6	20	10	17	6	4	9	7	19
8	HARIPUR	1019000	8	19	37	87	137	46	9	1	0	19	17	34
9	KARAK	763000	27	78	18	52	101	45	49	15	9	0	12	16
10	KOHAT	995000	489	595	317	836	149	98	40	24	33	20	42	40
11	KOHISTAN	480000	0	0	9	9	14	2	22	0	0	2	1	0
12	LOWER DIR	1307000	50	45	6	0	45	36	25	0	19	107	82	12
13	MALAKAND	815000	4	9	10	14	20	24	4	7	3	8	20	36
14	MANSEHRA	1760000	7	0	37	8	19	16	18	3	8	5	5	9
15	MARDAN	2478000	48	67	42	66	117	199	196	214	121	100	117	88
16	NOSHERA	1456000	10	4	87	2	16	27	65	6	134	36	7	8
17	PESHAWAR	3768000	146	106	46	53	114	294	97	246	38	144	48	62
18	SAWABI	1728000	64	81	67	94	156	59	153	98	101	129	108	339
19	SHANGLA	771000	20	4	13	19	15	13	179	10	418	12	4	8
20	TOOR GHAR	171395	7	7	0	4	5	13	11	17	13	24	31	21
21	UPPER DIR	936000	24	22	31	32	38	76	113	63	66	67	61	67
22	D.I. KHAN	1511000	212	418	598	410	440	165	328	148	131	146	262	346
23	LAKKI MARWAT	853000	16	59	219	167	257	56	30	136	24	0	199	10
24	SWAT	2271000	114	141	165	206	161	241	139	308	243	288	436	474
25	TANK	413000	79	75	65	12	17	24	11	7	4	6	5	5
